# Revising the definition of “demand satisfied for modern methods of family planning:” A cross-sectional study to explore incorporating person-centered constructs of demand, choice, and satisfaction

**DOI:** 10.1371/journal.pone.0316725

**Published:** 2025-01-06

**Authors:** Jewel Gausman, Niranjan Saggurti, Richard Adanu, Delia A. B. Bandoh, Mabel Berrueta, Suchandrima Chakraborty, Ernest Kenu, Nizamuddin Khan, Ana Langer, Carolina Nigri, Magdalene A. Odikro, Veronica Pingray, Sowmya Ramesh, Paula Vázquez, Caitlin R. Williams, R. Rima Jolivet

**Affiliations:** 1 Guttmacher Institute, New York, New York, United States of America; 2 Maternal and Child Health Nursing Department, School of Nursing, University of Jordan, Amman, Jordan; 3 Population Council, New Delhi, India; 4 Department of Population, Family, and Reproductive Health, University of Ghana School of Public Health, Accra, Ghana; 5 Department of Epidemiology and Disease Control, University of Ghana School of Public Health, Accra, Ghana; 6 Institute for Clinical Effectiveness and Health Policy, Buenos Aires, Argentina; 7 Department of Health Science, Kinesiology, and Rehabilitation, Universidad Nacional de La Matanza, Buenos Aires, Argentina; 8 Department of Maternal and Child Health, Gillings School of Global Public Health, University of North Carolina at Chapel Hill, Chapel Hill, North Carolina, United States of America; Ege University, School of Medicine, TÜRKIYE

## Abstract

Several challenges to validity have been identified with standard approaches used to measure “demand satisfied for modern methods of family planning.” This study explored construct validity of the widely used indicator for “demand satisfied” by comparing the standard definition to alternative definitions of the indicator highlighting dimensions of women’s own perceived demand, choice, and satisfaction. This cross-sectional study of women aged 15–49 years was conducted in Argentina (n = 1492), Ghana (n = 1600), and India (n = 1702) using a two-staged random sampling design. Women were directly asked about their: 1) demand, whether they wanted to use a contraceptive method to prevent pregnancy; 2) choice, whether they had autonomy in decision-making during their last family planning visit; and 3) satisfaction, whether they were satisfied with their method. The values of the standard and alternative indicators were compared. Convergent validity was assessed using logistic regression to explore the association between indicator definition and use of a preferred contraceptive method. In Argentina and India, the percentage of women with demand satisfied after incorporating constructs of demand, choice, and satisfaction was substantially lower than that obtained using the standard definition—a reduction of ~70% in Argentina and ~40% in India. Women who were categorized as having their “demand satisfied” for family planning according to the person-centered dimensions of the alternative indicator were significantly more likely to be using their preferred method of contraception in all three countries (OR: 7.7, 95% CI: 5.31–11.07 in Argentina, OR: 4.83, 95% CI: 2.27–10.27 in Ghana, and OR: 2.07 95% CI: 1.11–3.86 in India) compared to those whose demand was satisfied by only the standard indicator definition. Revising the definition of demand satisfied to reflect the principles of person-centered care offers an opportunity to improve construct validity by ensuring that global measurement efforts align with women’s reproductive rights.

## Introduction

Ensuring that women worldwide have access to high-quality family planning services and commodities is fundamental to improving maternal health. A 2012 Lancet report using World Health Organization maternal mortality estimates and data on contraceptive prevalence from the 2010 United Nations World Contraceptive Use database estimated that an additional 104,000 deaths per year could be averted by fulfilling unmet need for family planning. This represents a 29% annual global reduction in preventable maternal deaths [1]. In 2010, a global goal was set to enable 120 million more women and girls from the world’s poorest countries to voluntarily adopt modern contraception by 2020 [2]. Stakeholders prioritized “demand for family planning satisfied through modern methods of contraception” (hereafter, “demand satisfied”) as a core indicator for Strategies toward Ending Preventable Maternal Mortality (EPMM), the global strategic framework for maternal health in the Sustainable Development Goal (SDG) period, for its potential to drive progress in EPMM Key Theme #9, “address all causes of maternal mortality, reproductive and maternal morbidities, and related disabilities” [3]. Further, this indicator is tracked as SDG 3.7.1, the “proportion of women of reproductive age (aged 15–49 years) who have their need for family planning satisfied with modern methods.” Measuring “demand satisfied” is considered to be a key indicator to ensure universal access to sexual and reproductive health services and integration of reproductive health into national family planning strategies [4].

“Demand satisfied” is calculated by dividing the number of women who are currently using a modern method by the number of women who are using any method of contraception plus those who have an unmet need for family planning. Thus, the related indicator, “unmet need for family planning” (hereafter, “unmet need”) is a critical component of this measure. It is defined as the percentage of women of reproductive age who want to stop or delay childbearing but are not using any method of contraception. This indicator is typically calculated among women who are married/in-union or among women who are sexually active. These indicators therefore assign “demand” for family planning based on a woman’s responses to a set of questions about fertility intentions and current contraceptive use, rather than from a direct expression of her own perceived need or desire for contraception. Externally imposing assumptions about a woman’s demand for contraception runs counter to the core principles of person-centered care.

“Demand satisfied” and “unmet need” serve as cornerstones to track progress of global family planning programs and policy and to guide strategic investment; however, both indicators have been subject to criticism. The framing of latent contraceptive demand as a function of supply-side constraints through a macro-economic lens [5], rather than one that prioritizes women’s own subjective demand, runs contrary to recent efforts to emphasize person-centered care in global reproductive health programming. Respectful, person-centered care that is responsive to individual needs, preferences, and values is a fundamental aspect of care quality [6, 7], and ensuring that family planning services incorporate a rights-based perspective has been identified as a global priority [8–11].

How well the derived measure of demand correlates with women’s own subjective perceptions of their demand for contraception is arguable [12]. Previous research has found that a substantial proportion of women defined as having an “unmet need” have no intention to use contraception in the future [13]. The measure’s emphasis on a supply-side, systems perspective may not fully reflect the most important reasons for unfulfilled demand from an individual perspective [14]. Few women indicate that lack of access is the underlying cause of their nonuse; rather fears over side-effects and infrequent sex are more frequently cited drivers of nonuse [15]. Up to 73% of women defined as having unmet need in Nepal and 58% in Bangladesh have cited infrequent sexual intercourse as a primary reason for contraceptive nonuse [16]. Furthermore, by not incorporating individual perceptions of risk, the standard formulation of the indicator imposes gendered assumptions about sexual activity and risk that reflect outdated norms, which may disempower and distort contraceptive demand among unmarried women and sexual minorities. Moreau and colleagues proposed two new measures to estimate a woman’s unmet need and unmet demand that address some of these challenges with the current indicator. In the former, the authors refine the construct of risk to exclude women who are not sexually active whereas the latter identifies women with unmet need who state a desire to use contraception in the future, signaling unmet stated demand [17].

In addition to demand, the construct of choice is also fundamental to global family planning efforts and is a construct that is rooted in the principles of patient-centered care and basic human rights [18–20]. Informed choice has been operationalized in family planning programs as enabling women to choose the best method for them based on a thorough understanding of their contraceptive options [21]. However, the line between a choice driven by a client and that driven by a provider is sometimes murky in the face of a woman’s specific health circumstances, contraindications, and method effectiveness. While giving women all the information about all of the methods in order to provide her with the opportunity to make a fully autonomous decision that is medically sound is a difficult goal to realize [21], provider-directed contraceptive counseling, whereby the provider may recommend certain methods based on assumptions related to women’s preferences and method efficacy, comes with some concerns in relation to reproductive justice and coercion [22]. In light of these challenges, another way to operationalize choice in the context of family planning may be to examine whether women’s experience of care reflected an autonomous decision-making process. The “Mothers Autonomy in Decision-Making (MADM)” scale was designed to measure autonomy in decision-making during maternity care, defined as a person’s ability to lead decision-making related to labor and childbirth [23]. A similar scale has been proposed and validated in the domain of family planning [24] called the Family Planning Autonomous Decision-Making (FP-ADM) scale, which was adapted from the MADM scale [23].

Finally, ensuring that women choose and use the “right” method for them is fundamental to the idea of “demand satisfied,” though these elements of personal satisfaction are not captured in the measure. Specifically, it has been argued that women currently using contraception may have an “unmet need” if they are not using a method that meets their reproductive needs [25]. Positive experiences of care are an indivisible element of quality, equal in importance to the provision of scientifically sound, medically indicated care interventions in the current World Health Organization definition of quality of care [26]. Not capturing the dimension of personal satisfaction arguably underestimates the number of women with the unmet need when defining the construct from a person-centered perspective. Others have argued that questions should be added to the DHS survey to incorporate method satisfaction into the definition of unmet need [27], though a strategy for recalculating the indicator has not been proposed. Including a more direct measure of individual satisfaction would align with the concept of person-centered care by elevating the importance of individual preferences in calculating the indicator while making the indicator more consistent with a rights-based approach.

Construct validity can be defined as “accuracy of the operationalization of a concept or phenomenon” [28]. Through this lens, a person-centered measure of “demand satisfied” would enable women themselves to register their demand for contraception, operationalize the human rights principles of self-determination and decisional autonomy [7], and track whether women are satisfied with the method that they ultimately choose. Thus, this study aimed to explore construct validity of the widely used “demand satisfied” indicator by comparing results of the standard definition used in global measurement to alternative definitions of the indicator incorporating dimensions of women’s own perceived demand, choice, and satisfaction.

## Methods

### Study description

This cross-sectional study included women aged 15–49 years and took place in four discrete, subnational areas in Argentina, Ghana, and India. More details on selection of the geographic areas can be found in the published study protocol [29].

### Participants

In each study area, 20 primary sampling units were randomly selected based on probability proportional to size. The sampling frame was adapted from the Demographic and Health Surveys in Ghana and India and the Multiple Indicator Cluster Survey in Argentina. In Ghana and India, community mapping identified households with women of reproductive age (15–49 years) in each primary sampling unit. Out of this list, a minimum of 18 women from different households in each primary sampling unit were randomly selected, for a total of at least 360 women per district and 1440 women per country. In Argentina, interviews with a random sample of 360 women per primary sampling unit were conducted upon exit from facilities providing reproductive and maternal health services, as conducting household surveys was not feasible given low population density. Interviews were conducted between October 2020 through December 2020 in Ghana, October 2020 through March 2021 in India, and March 2021 through June 2021 in Argentina.

Women were included in the study if they agreed to participate and if they were aged 15–49 years at the time of the study. Written informed consent was obtained from participants ≥18 years old in Ghana and India and ≥ 16 years old in Argentina; parental permission and written informed assent was obtained for minors <18 years old in Ghana and India and < 16 years old in Argentina.

### Data collection

To collect data on contraceptive demand, choice, and satisfaction, women were asked a series of direct questions regarding: 1) demand, whether they wanted to use a contraceptive method to prevent pregnancy; 2) choice, whether they had a high degree of autonomy in decision-making during their last family planning visit; and 3) satisfaction, whether they were satisfied with their family planning method and quality of care. Data on demand was captured by a single question with a “yes/no” response. All non-pregnant women were asked “Do you want/desire to be using a method of contraception right now?” while pregnant women were asked, “After your current pregnancy, will you want/desire to use a method of contraception so you don’t get pregnant again immediately?” Women’s autonomy in decision-making was assessed with the Family Planning Autonomy in Decision-Making (FP-ADM) scale. FP-ADM is a validated measure that focuses on autonomy surrounding a woman’s decision to use a contraceptive method within the context of contraceptive counselling [24]. Finally, data on satisfaction was assessed by a single “yes/no” question, “Are you satisfied with the method you are currently using?”

To collect data to calculate “demand satisfied” according to the standard definition, the same women were asked the standard DHS questions used to calculate the indicator. Basic demographic data were collected on participating women, including wealth quintile, education, literacy, age, marital status, and place of residence. Wealth quintile was calculated by administering the country-specific EquityTool module [30].

### Analysis

#### Validating the assumptions of the standard indicator

The standard “demand satisfied” indicator is defined as the number of women currently using any modern contraceptive method divided by the number of women who are using any method of contraception plus women who are considered to have an “unmet need” [31]. In concept, women with “unmet need” are those who are fecund and married/in-union or sexually active, but who are not using a method of contraception, and say that the do not want any more children or that they want to delay their next child. Currently pregnant and post-partum women are considered to have unmet need if their current or last pregnancy was “not wanted” or mistimed. The indicator is typically calculated among two populations: 1) women who are married/in-union and 2) women who are considered to be sexually active. Women are automatically considered to be sexually active if they are married or in-union. Unmarried women are considered to be sexually active if they report having had sexual intercourse within the last 30 days. Extensive details on how to calculate the value of the indicator are published elsewhere [32].

The standard indicator makes several assumptions inherent to the underlying constructs of demand, choice, and satisfaction. A woman who is sexually active or married/in-union and does not want to have a child in the next two years and is not using a modern method of family planning is assumed to have unmet need (thus, demand) for family planning. To examine the validity of this assumption, we compare women’s stated demand for family planning with their derived unmet need. The standard indicator also makes assumptions about latent contraceptive demand for unmarried women based on a woman’s retrospective report of whether she has been sexually active in the previous 30 days. We explore the validity of this assumption by tabulating women’s stated demand for family planning according to their sexual activity. Finally, we explore how the standard indicator correlates with the constructs of choice and satisfaction by examining the percentage of women who are considered by the standard indicator as having their “demand satisfied” who 1) had high decisional autonomy at their last family planning visit and 2) indicated that they were satisfied with their family planning method.

#### Comparing the standard indicator measuring “demand satisfied” to the alternative person-centered formulation

To compare the standard definition to alternative definitions of “demand satisfied,” we iteratively redefined the numerator and denominator of the indicator to include women’s direct perspectives on demand, choice, and satisfaction. The standard and alternative definitions of the indicators used in this study are provided in **Table 1**.

**Table 1:** Standard and alternative indicator definitions.

Definitions			
**Standard Indicator**	**Numerator**	**Denominator**	**Population**
Demand for modern methods satisfied among sexually active women	Women using a modern method of family planning	Women with unmet need for family planning + women using any method of family planning	Sexually active women between ages 15 and 49
Demand for modern methods satisfied among married or in-union women	Women using a modern method of family planning	Women with unmet need for family planning + women using any method of family planning	Married or in-union women between ages 15 and 49
**Alternative Formulations**	** **	** **	** **
Demand for modern methods met	Women using a modern method of family planning	Women who say they want to use a FP method now or after current pregnancy + women using any method of family planning	All women
Demand for modern methods met with high decisional autonomy	Women using a modern method of family planning AND who have FP-ADM scores >2	Women who say they want to use a FP method now or after current pregnancy + women using any method of family planning	All women
Demand for modern methods met with high method satisfaction	Women using a modern method of family planning AND who are satisfied with their current method	Women who say they want to use a FP method now or after current pregnancy + women using any method of family planning	All women
Demand for modern methods met with high decisional autonomy and method satisfaction	Women using a modern method of family planning AND who have FP-ADM scores >2 AND who are satisfied with their current method	Women who say they want to use a FP method now or after current pregnancy + women using any method of family planning	All women

The alternative definitions of the indicator rely on women’s direct reports. Unlike the standard indicator, we included all woman with a stated demand for family planning (either now or after their current pregnancy) in the denominator. We did not differentiate women based on marital status or past sexual activity; rather, women were included based on their own personal desire to use a method of contraception. In the numerator, we iteratively incorporated the elements of choice and satisfaction. To reflect choice, women were considered to have high decisional autonomy if they had an FP-ADM score >2 (out of a possible score of three) during their last family planning visit. The construct of satisfaction was measured by asking women directly whether they were satisfied with their current method.

We calculated and compared the standard indicator to the alternative formulation. We then used alluvial plots to evaluate how specific populations of women defined by the current indicator are classified in the alternative measure. Last, we assessed convergent validity by evaluating the association between women who are categorized as having their demand satisfied by only the standard indicator versus those who have their demand satisfied defined by the alternative, person-centered indicator. Conceptually, we expected that women whose demand is satisfied by the alternative, person-centered indicator would have higher odds of stating they are using their preferred method of contraception.

### Ethics approval

The Institutional Review Board (IRB) of the Harvard T.H. Chan School of Public Health approved this study on 4 September 2019. The research is classified as Level 4 Data using Harvard’s Data Security Policy. The study also was approved in Argentina by the Comité de Ética de la Investigación de la Provincia de Jujuy (approval ID not applicable), Comisión Provincial de Investigaciones Biomédicas de la Provincia de Salta (approval ID: 321-284616/2019), Consejo Provincial de Bioética de la Provincia de La Pampa (approval ID not applicable), and Comité de Ética Central de la Provincia de Buenos Aires (approval ID: 2919-2056-2019); in Ghana by the Ghana Health Service Ethical Review Board (approval ID: GHS-ERC022/08/19); and in India by the national population council IRB (approval ID: 889) and local Sigma-IRB (approval ID: 10052/IRB/19-20).

## Results

The study sample is described in **Table 2**. Response rates were high in all three countries (100% in Argentina and Ghana, and 93% in India). In Argentina and India, the majority of participants were literate and had completed at least secondary education. However, in India, a sizeable minority of the population (23.3%) was unable to read or write or could only sign their name. In Ghana, half of the participants were unable to read or write (52.6%) and over a third had no formal education (37.8%). Compared to the national average, the study population in Argentina tended to come from the middle three wealth quintiles. In Ghana, the sample tended to exhibit a higher degree of poverty compared to the national average in other countries, with 29.8% of the sample coming from the poorest wealth quintile. In India, the sample tended to be wealthier, with nearly 40% of the study population coming from the wealthiest quintile. In all countries, most women were married or in a stable union at the time of data collection, although 17.9% of the sample in Argentina, 30.3% in Ghana, and 7.5% in India were never married.

**Table 2:** Sample description.

	Argentina	Ghana	India
	% (n)	% (n)	% (n)
n	1492	1600	1702
Age			
15–19 years	10.25 (153)	12.6 (201)	5.99 (102)
20–24 years	27.14 (405)	20.6 (329)	17.63 (300)
25–29 years	26.27 (392)	21.5 (344)	22.33 (380)
30–39 years	27.08 (404)	25.7 (411)	36.72 (625)
≥40 years	9.25 (138)	10.6 (169)	17.33 (295)
Don’t know	0.00 (0)	8.8 (140)	0.0 (0)
Refused	0.00 (0)	0.4 (6)	0.0 (0)
Literacy level			
Read and write	98.46 (1469)	43.0 (688)	76.15 (1296)
Read only	0.87 (13)	2.4 (39)	0.35 (6)
Can sign name only	0.34 (5)	1.6 (25)	10.52 (179)
Cannot read and write	0.00 (0)	52.6 (842)	12.75 (217)
Don’t know	0.00 (0)	0.3 (4)	0.12 (4)
Refused	0.00 (0)	0.1 (2)	0.0 (0)
Highest level of education completed			
No formal education	0.60 (9)	37.8 (604)	9.32 (138)
Primary/elementary	23.93 (357)	32 (511)	35.11 (520)
Secondary	57.57 (859)	23.4 (373)	37.68 (558)
Technical/vocational	1.07 (16)	1.9 (30)	1.82 (27)
Tertiary/college	16.09 (240)	3.7 (59)	3.78 (56)
Graduate/professional degree	0.27 (4)	0.7 (11)	11.07 (164)
Other	0.07 (1)	0.4 (7)	0.74 (11)
Don’t know	0.00 (0)	0.1 (1)	0.47 (7)
Refused	0.00 (0)	0.0 (0)	0.0 (0)
Wealth quintile			
Poorest	7.24 (108)	29.8 (473)	1.0 (17)
Poor	20.64 (308)	22 (349)	5.06 (86)
Middle	29.62 (442)	19.5 (310)	17.25 (293)
Rich	23.59 (352)	12.7 (202)	37.08 (630)
Richest	6.50 (97)	16 (255)	39.61 (673)
Marital status			
Currently married/in stable union	76.75 (1145)	65.2 (1042)	89.9 (1529)
Widowed	0.47 (7)	1.5 (24)	2 (34)
Divorced/separated	4.29 (64)	3.1 (49)	0.6 (11)
Never married/Single	17.90 (267)	30.3 (484)	7.5 (128)
Refused or Missing	0.60 (9)	0.1 (1)	0.0 (0)
Currently pregnant			
Yes	23.93 (357)	13.73 (197)	5.93 (101)
No	75.34 (1,124)	86.27 (1238)	86.06 (1465)
Don’t know or Missing	0.74 (11)	10.31 (165)	7.99 (136)

Current use of contraception among non-pregnant women in the study is described in **Table 3**. Argentina had the highest contraceptive prevalence with 76.3% of women using a method, compared to 29.3% in Ghana and 59.3% in India. The oral contraceptive pill and male condom were the most common methods used in Argentina (25.9% and 21.4%, respectively). In Ghana, the majority of contraceptive users (46.2%) were using injectables, while in India female sterilization (50.0%) and male condom (33.1%) were the most frequently used methods.

**Table 3:** Current use of contraception among non-pregnant women.

	Argentina	Ghana	India
	% (n)	% (n)	% (n)
	1124	1238	1465
Current use of contraception (any method)			
Yes	76.32 (722)	29.24 (362)	59.39 (870)
No	18.50 (175)	70.76 (876)	40.61 (595)
Don’t know	0.0 (0)	0.0 (0)	0.0 (0)
Refused	1.16 (13)	0.0 (0)	0.0 (0)
Current method*			
Female sterilization	15.62 (124)	0.29 (1)	49.95 (465)
Male sterilization	0.00 (0)	0.0 (0)	0.0 (0)
IUD	16.75 (133)	2.60(9)	6.44 (60)
Implant	13.98 (111)	19.65 (68)	0.0 (0)
Injectables	13.85 (110)	46.24 (160)	2.47 (23)
Pill	25.94 (206)	11.56 (40)	7.2 (67)
Male condom	21.41 (170)	6.94 (24)	33.08 (308)
Female condom	0.00 (0)	0.0 (0)	0.54 (5)
Emergency contraception	0.50 (4)	3.18 (11)	0.43 (4)
Diaphragm	0.00 (0)	0.0 (0)	0.21 (2)
Foam/jelly	0.00 (0)	0.0 (0)	0.97 (9)
Contraceptive patch	0.38 (3)	0.0 (0)	0.0 (0)
Contraceptive vagina ring	0.00 (0)	0.0 (0)	0.0 (0)
Lactational amenorrhea method	2.77 (22)	2.31 (8)	0.11 (1)
Other modern method	0.88 (7)	0.29 (1)	0.43 (4)
Standard days method	0.88 (7)	10.12 (35)	1.18 (11)
Withdrawal	1.64 (13)	1.73 (6)	2.58 (24)
Fertility awareness/periodic abstinence	0.13 (1)	1.45 (5)	0.21 (2)
Other traditional method	0.00 (0)	0.0 (0)	0.11 (1)
Don’t know	0.13 (1)	0.0 (0)	0.0 (0)
Refused	1.13 (9)	0.29 (1)	0.0 (0)

*May add up to more than 100% because respondents could select multiple methods.

Unmet need (as defined through the standard definition) varied across and within countries (**Table 4**). Unmet need was highest in Ghana, estimated at 29.6% among married women and 31.8% among sexually active women. In Ghana, among both married and sexually active women, there were substantial differences in unmet need between districts. Overall, unmet need tended to be slightly higher among sexually active women compared to married women. In India, estimates for unmet need were identical when calculated among women who are married/in a union and among sexually active women because all women in the sample who were never-married stated that they were not sexually active.

**Table 4:** Unmet need for family planning among married and sexually active women in subnational study areas of Argentina, Ghana, and India.

	Unmet need (married women)		Unmet need (sexually active women)	
	% Yes (n)	% No (n)	% Yes (n)	% No (n)
Argentina	176	969	197	1062
Buenos Aires	13.76 (41)	86.24 (257)	13.31 (43)	86.69 (280)
Jujuy	18.82 (54)	81.18 (233)	19.80 (60)	80.20 (243)
La Pampa	10.60 (30)	89.40 (253)	11.01 (36)	88.99 (291)
Salta	18.41 (51)	81.56 (226)	18.95 (58)	81.05 (248)
Total	15.37 (176)	84.63 (969)	15.65 (197)	84.35 (1062)
Ghana	308	734	383	820
Bunkpurugu Yunyoo	23.5 (78)	76.5 (254)	25.21 (89)	74.79 (265)
Tolon	35.94 (124)	64.1 (221)	36.31 (130)	63.69 (228)
Sunyani Municipal	19.77 (34)	80.23 (138)	21.79 (51)	78.21 (183)
Techiman North	37.31 (72)	62.69 (121)	43.80 (113)	56.20 (145)
Total	29.6 (308)	70.44 (734)	31.84 (383)	68.16 (820)
India	147	1382	147	1383
Meerut	4.40 (18)	95.60 (391)	4.40 (18)	95.60 (391)
Gonda	16.34 (66)	83.66 (338)	16.34 (66)	83.66 (338)
Tiruvallur	8.43 (29)	91.57 (315)	8.43 (29)	91.57 (315)
Krishnagiri	9.12 (34)	90.88 (339)	9.12 (34)	90.88 (339)
Total	9.61 (147)	90.39 (1383)	9.61 (147)	90.39 (1383)

**Fig 1:**
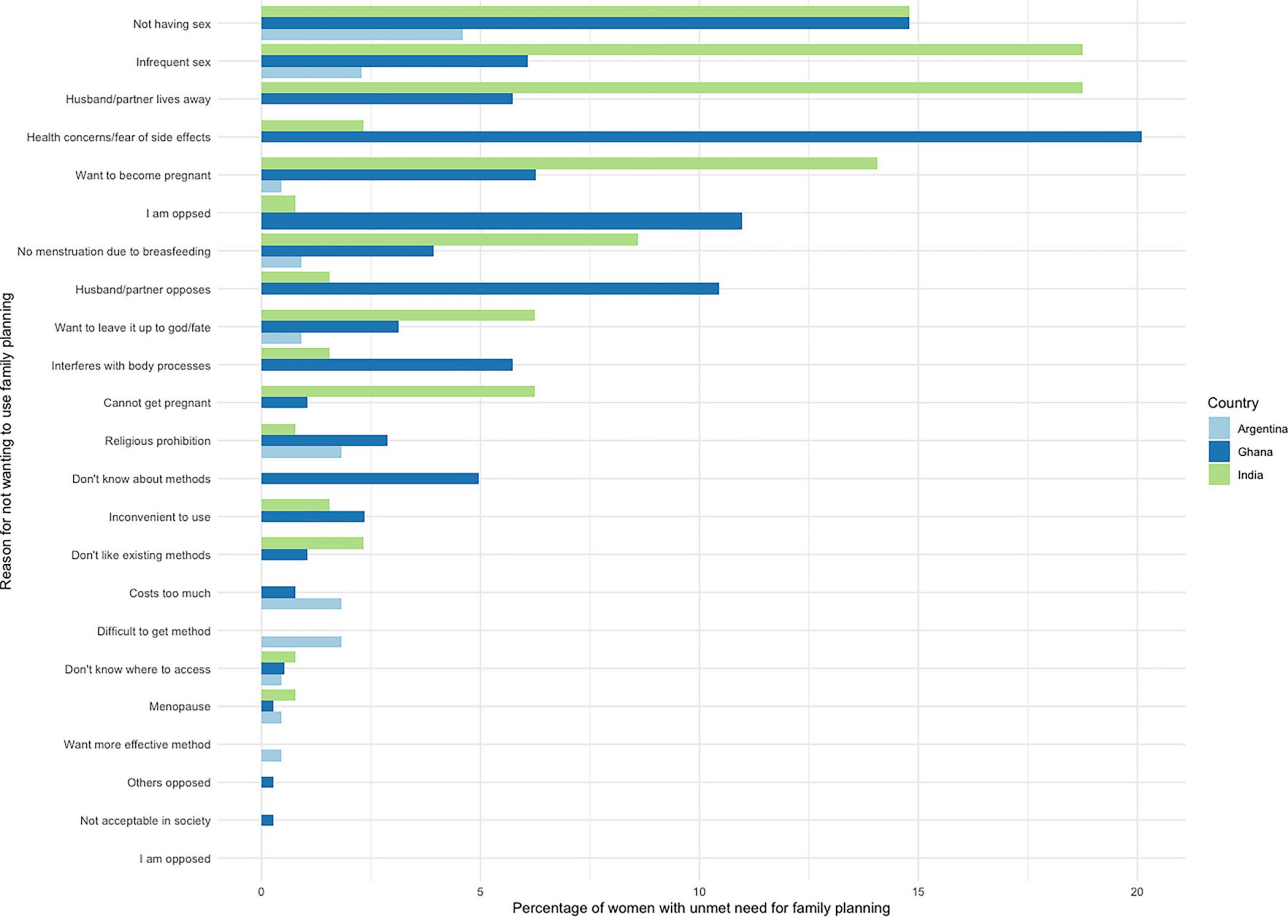
Reasons women with unmet need are not using a family planning method in Argentina, Ghana, and India.

Unmet need did not correspond with women’s stated demand for family planning. In Ghana and India, most women who were categorized as having unmet need for family planning indicated that they did not want to use a method. Only 25.0% of women with unmet need in India and 26.1% in Ghana stated that they wanted to use a family planning method. In Argentina, the percentage was higher, although only 61.3% of women considered to have unmet need stated that they wanted to be using a family planning method when asked directly. **Fig 1** shows the reasons why non-pregnant women considered to have having an unmet need did not want to use family planning.

A substantial proportion of women who were not sexually active stated that they wanted to use a method of family planning when asked directly. In Argentina, 61.2% (n = 118) of women who were single (not married or in-union) and not sexually active stated that they wanted to be using a method of family planning. The percentages of single women who stated having a demand for family planning but were not sexually active in Ghana and India were also significant: 24.0% (n = 92) in Ghana and 19.2% (n = 33) in India. Of note, in all countries, a substantial proportion of women who were married or in-union stated that they were not sexually active: 37.8% (n = 433) in Argentina, 42.2% (n = 440) in Ghana, and 28.0% (n = 428) in India.

**Fig 2:**
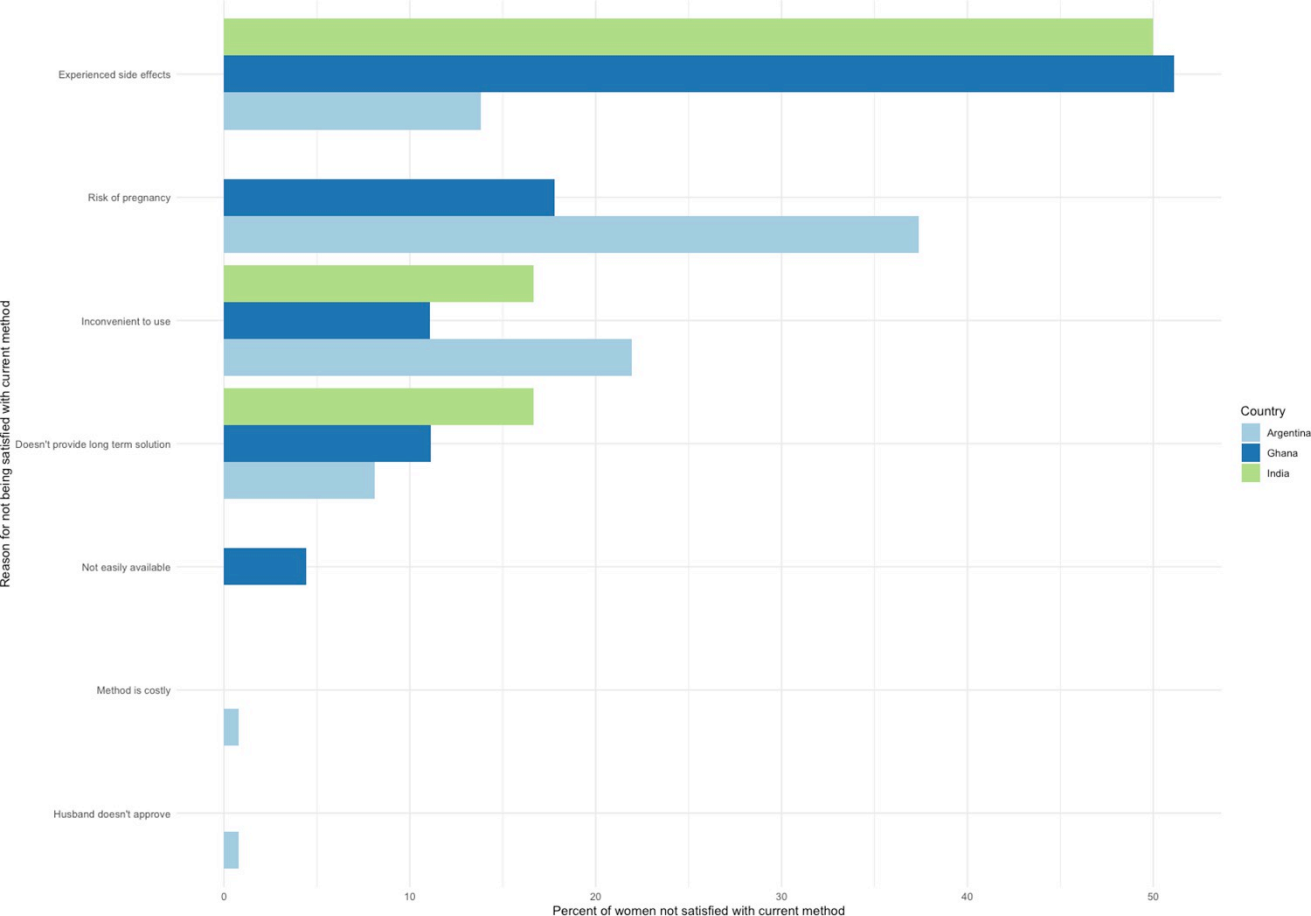
Reasons for dissatisfaction with current family planning method among women in Argentina, Ghana, and India.

Decisional autonomy and stated satisfaction among women who are defined as having their “demand satisfied,” varies across study settings (**Table 5).** More than 90% of women in Argentina and Ghana, and only ~70% in India, categorized as having their demand for family planning satisfied as per the standard indicator reported high decisional autonomy during their last family planning visit. Most women categorized as having their demand for family planning satisfied also stated being satisfied with their current method, however >10% of women in Argentina and Ghana—a significant minority—indicated a lack of satisfaction. **Fig 2** shows the reasons why women in each country were not satisfied with their current method. In India and Ghana, most women who were unsatisfied with their current method were unhappy due to experienced side effects, while in Argentina, women expressed dissatisfaction with the risk of pregnancy and inconvenience associated with their current method.

**Table 5:** Decisional autonomy and method satisfaction among women whose demand for modern family planning is considered to be satisfied per the standard indicator.

	High level of decisional autonomy at last visit (FP-ADM >2)		Satisfied with current method		Total, % (n)
	Yes % (n)	No % (n)	Yes % (n)	No % (n)	
Percent of women with demand satisfied (among women married/in a union)					
Argentina	92.37 (351)	7.63 (29)	82.96 (66)	13.39 (66)	100 (380)
Ghana	95.78 (227)	4.22 (10)	87.12 (203)	12.88 (30)	100 (237)
India	69.94 (556)	30.06 (239)	99.27 (819)	0.48 (4)	100 (795)
Percent of women with demand satisfied (among sexually active women)					
Argentina	91.88 (396)	8.12 (35)	82.80 (462)	13.44 (75)	100 (431)
Ghana	95.92 (282)	4.08 (12)	88.74 (260)	11.26 (11)	100 (294)
India	69.94 (556)	30.06 (239)	99.27 (819)	0.48 (4)	100 (795)

**Fig 3:**
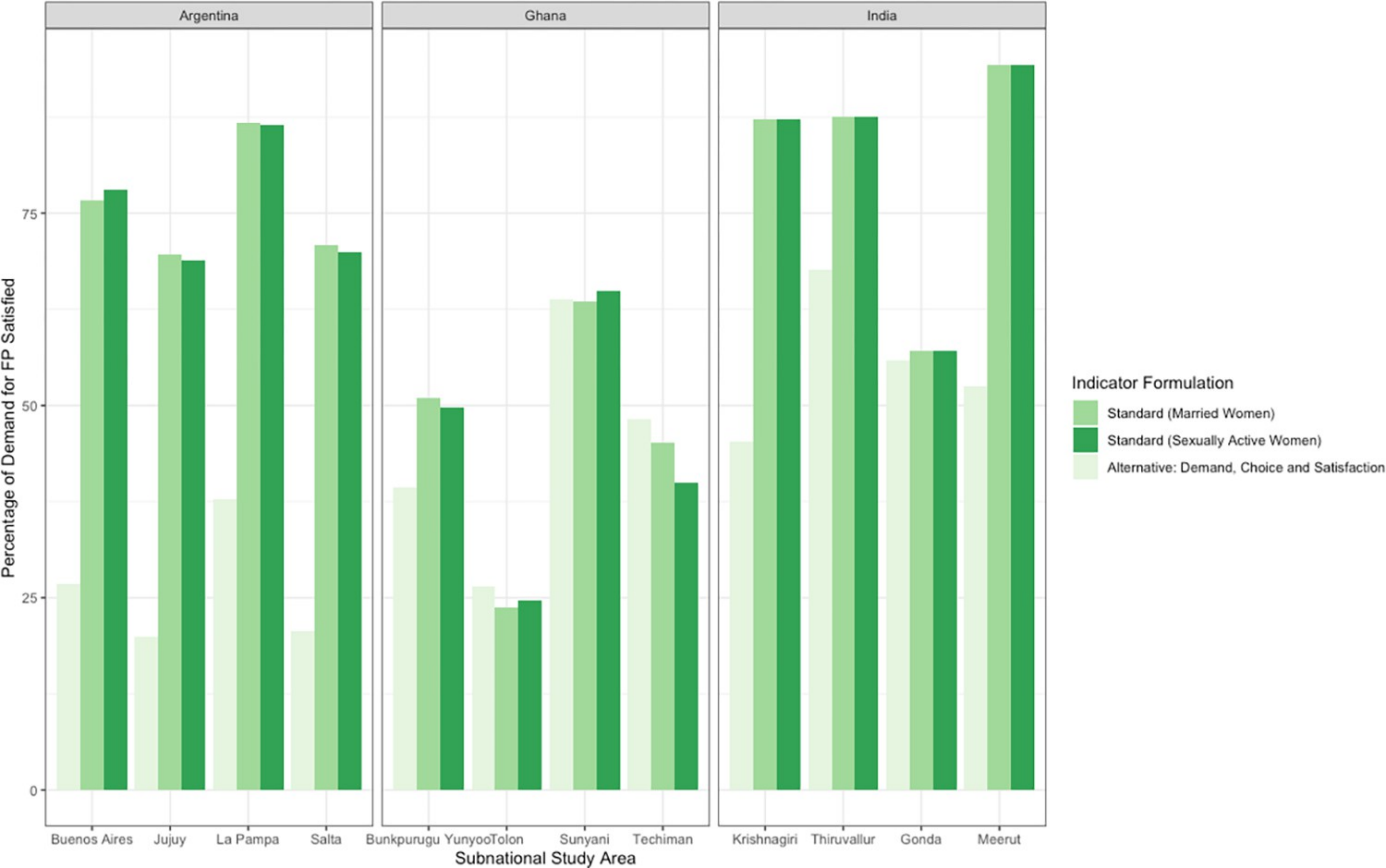
Married and sexually active women with demand satisfied for family planning per the standard indicator versus the alternative indicator incorporating demand, choice, and satisfaction in subnational study areas in Argentina, Ghana, and India.

The value of the alternative indicator compared to the standard is substantially lower in Argentina and India, but roughly the same in Ghana (Table 6). In Argentina, 76.8% of sexually active women and 76.7% of married women had their demand for modern family planning satisfied per the standard indicator. The value of the indicator decreased to 24.0% after incorporating the three constructs of demand, choice, and satisfaction in the alternative definition. A similar reduction in the value of the alternative indicator incorporating all three person-centered constructs was also observed in India, from 85.0% according to the standard indicator to 53.0% per the alternative definition. In Ghana, there was little change in the indicator’s value between standard and alternative definitions (45.2% per the alternative indicator compared to 43.4% among sexually active women 43.7% among married women as per the standard indicator). Subnational estimates comparing standard and alternative definitions of the indicator are provided in **Fig 3** for each study country. In Ghana, the value of the alternative indicator was higher in all subnational study areas except for Bunkpurugu Yunyoo and Sunyani (among sexually active women only).

**Table 6:** Comparison of standard and alternative definitions of the indicator to measure "demand satisfied".

	Argentina			Ghana			India		
Definitions	Numerator	Denominator	Value	Numerator	Denominator	Value	Numerator	Denominator	Value
**Standard Indicator**									
Demand for modern methods satisfied among sexually active women	671	874	76.77	303	692	43.4	869	1022	85.03
Demand for modern methods satisfied among married women	597	779	76.64	241	555	43.7	869	1022	85.03
**Alternative Formulations**									
Demand for modern methods met	744	1316	56.53	360	639	56.34	893	1020	87.55
Demand for modern methods met with high decisional autonomy	447	1316	33.97	331	639	51.80	571	1020	55.98
Demand for modern methods met with high method satisfaction	506	1316	38.45	302	639	47.26	830	1020	81.37
Demand for modern methods met with high decisional autonomy and method satisfaction	316	1316	24.01	289	639	45.23	541	1020	53.04

Argentina observed the largest changes in the denominator when comparing the standard and alternative formulations of the indicator, from 874 sexually active women (197 women with unmet need and 677 women using any method of contraception) and 779 married or in-union women (176 women with unmet need and 603 women using any method of contraception) using the standard definition to 1316 women using the alternative definition. In Ghana and India, the changes observed in the denominator were minimal. In Argentina and India, the number of women in the numerator decreases substantially when comparing the standard and alternative definition. In Argentina, adding in the constructs of decisional autonomy and satisfaction individually each reduced the numerator by about 20 percentage points. In India, the dramatic decrease in the numerator was observed only when adding in the construct of decisional autonomy, and a reduction by only six percentage points was observed in relation to method satisfaction.

**Fig 4:**
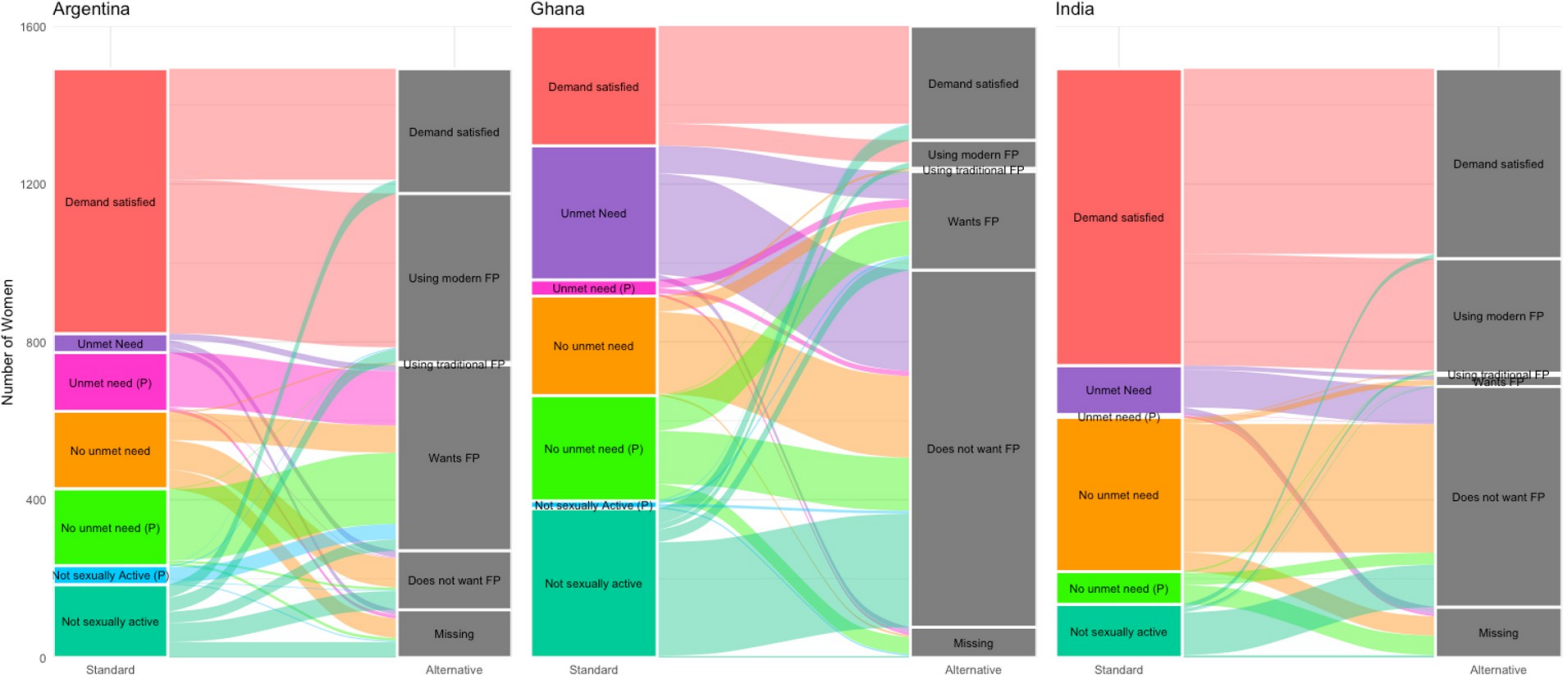
Alluvial diagrams illustrating the categorization of women defined in the standard versus alternative indicator in Argentina, Ghana, and India.

The alluvial diagrams presented in **Fig 4** show in detail how women are categorized per the standard and alternative indicators. In Argentina, the large increase in the denominator relates to the substantial proportion of women categorized as having no unmet need who nevertheless express a desire to use a method of contraception, while also drawing from the population of women who are not included in the standard indicator because they are not considered to be sexually active. In Ghana and India, the alluvial plots illustrate that while women categorized as having unmet need per the standard indicator with a stated lack of desire to use family planning are removed from the denominator in the alterative formulation of the indicator, the alternative denominator then includes women who stated wanting to use a method of family planning although not considered to be sexually active or to have any unmet need. The misalignment of women in the denominator between the two formulations of the indicator is especially prominent among pregnant and post-partum amenorrheic women. While an important percentage of women in all three countries who are considered to have unmet need per the standard formulation (in fact, more than 75% in India and Ghana) stated not wanting to use a method of contraception, there was greater alignment between the standard and alternative formulations with regard to pregnant/post-partum amenorrheic women, as a substantial percentage of pregnant/post-partum amenorrheic with unmet need wanted to use a method of family planning now or at the end of their pregnancy. However, among pregnant/post-partum amenorrheic women deemed to have no unmet need by the standard indicator, our results revealed that a sizeable percentage of these women stated a desire to use family planning. In Argentina, this was particularly visible where 92.3% (n = 195) of pregnant/post-partum amenorrheic women with no unmet need stated that they desire to use a method of contraception. Further, in Argentina and Ghana, 3.2% (n = 48) and 1.1% (n = 18) of women who were pregnant/post-partum amenorrheic were not considered to be sexually active as defined by the standard indicator, due to the fact that they were not married or in-union and had not reported sexual activity in the last 30 days. A substantial percentage of these women (and in Argentina, a large majority) stated a desire to use family planning now or after their current pregnancy.

In all three countries, women categorized as having “demand satisfied” only through the standard indicator were significantly less likely to be using their preferred method of contraception compared to women who whose contraceptive demands were satisfied according to the person-centered measure (**Table 7)**. Logistic regression suggested that in Argentina, women who had their contraceptive demand satisfied according to the alternative indicator had 7.7 times the odds (95% CI: 5.37–11.07, p<0.0001) of using their preferred method of contraception compared to women whose demand was only satisfied according to the standard indicator. While the magnitude of the coefficient was greatest in Argentina, the statistical association was significant and in the expected direction in both Ghana and India.

**Table 7:** Simple logistic regression of different definitions of the indicator "demand satisfied" for women’s use of preferred contraceptive method (among women currently using any method).

	Women using preferred method of contraception								
	Argentina			Ghana			India		
**Women have demand for modern family planning satisfied according to:**	% Yes (n)	OR (95% CI)	p-value	% Yes (n)	OR (95% CI)	p-value	% Yes (n)	OR (95% CI)	p-value
Standard Indicator Only (reference)									
Alternative (person-centered) Indicator	40.26 (157)	1 (1–1)		69.57 (32)	1 (1–1)		92.64 (277)	1 (1–1)	
	83.86 (265)	7.71 (5.37–11.07)	<0.0001	91.70 (265)	4.83 (2.27–10.27)	<0.0001	96.30 (521)	2.07 (1.11–3.86)	0.022

## Discussion

Our results challenge several assumptions in measurement related to assumed versus perceived demand for family planning, and satisfaction, that have concrete implications related to contraceptive policy, planning and service delivery. The alternative indicator to measure “demand satisfied” from a person-centered perspective proposed here provides new data in support of operationalizing such principles in measurement, and reframes this widely used global indicator from its demographic origins toward a human-rights perspective.

In counterbalance to the ambitious FP2020 (now FP2030) targets for contraceptive prevalence from the 2012 London Family Planning Summit [33], health and human rights organizations and civil society actors have called for reinforcement of the rights-based approach that was the platform of the 1994 International Conference on Population and Development and reaffirmed in 2013 [34]. There are many calls to ensure that fundamental human rights principles grounded in respect for individual autonomy, values, and preferences—including agency, transparency, informed choice, participation and voice, and accountability [35]—guide access to contraception information and service delivery [10, 19, 26, 36, 37]. Our results provide concrete evidence for variation in the value of an indicator to measure “demand satisfied” from a woman’s perspective compared to the standard supply-side approach. In two of the three countries in this study, the standard measure of “demand satisfied” substantially overstates progress achieved in meeting the contraceptive needs of women when compared to the value of the alternative indicator proposed.

Unmet need has long been equated with latent demand for family planning [38, 39], an assumption that has been criticized for failing to recognize women’s autonomy in decision-making regarding fertility by prioritizing supply-side barriers and discounting women’s own reasons for contraceptive demand, or a lack thereof [40]. Redefining the denominator to incorporate direct, rather than construed, demand reflects important changes in how demand for family planning is defined in measurement. In Argentina, the denominator expands considerably in the alternative formulation that include all women who indicated that they wanted to be using a modern family planning method, regardless of marital status or stated sexual activity. While there is little change in the value of the denominator in Ghana and India, we argue that the alternative version of the indicator corrects the misclassification that results in using unmet need as a proxy for demand.

Along these lines, we argue that the standard indicator discounts women’s sexual agency and represents outdated social norms, which is evidenced by our data. The standard indicator’s assumption that all married women are at risk of unintended pregnancy fails to account for women’s actual sexual behavior. Globally, a large percentage of women in marriage or stable unions report not having been exposed to sexual intercourse during the last month; by the same token, a large percentage of unmarried women report having had recent sexual intercourse [41]. Data from our study reaffirms these findings and suggests that marital status is likely a poor proxy for sexual exposure and thus, risk of pregnancy. To address this issue, other alternative indicators have been proposed to measure “demand satisfied” by anchoring unmet need to past sexual exposure [17]. There may be additional challenges with this approach, especially in measuring unmet need among unmarried women, where social stigma towards unmarried women engaging in sexual activity is high [42–44]. To address these concerns, we shift our focus away from using a retrospective assessment of sexual exposure as a proxy of demand for contraception by asking women prospectively if they would like to use a method of family planning now, or after their current pregnancy. It is an important reflection of informed choice that a provider work with clients in order to support them in choosing the best approach for their health by weighing the potential risks and benefits. We believe that taking into account each woman’s perspective about whether she wants to use a contraceptive method better accounts for women’s anticipated risk of unintended pregnancy and their reproductive autonomy by allowing women who are unmarried and have not had recent sexual intercourse to register a demand for family planning. The state of being sexually active is not static; women who abstain from sex for a specific period of time may plan to resume or initiate sexual activity in the near future—a transition that may not be entirely predictable. Such women may want to start a method of family planning to protect against the possibility of a future risk of pregnancy, something that is not considered in the standard indicator definition. Furthermore, women who are not sexually active may want to use contraception for a variety of non-contraceptive purposes, including regulation of menstrual irregularity and/or pain [45]. By the same token, we believe that using a woman’s stated demand for family planning allows sexual minority women who may not be risk of pregnancy from sexual intercourse to be excluded if that better represents their desire.

The fact that more pregnant and post-partum women are included in the indicator’s denominator when “demand satisfied” is defined from a person-centered perspective than are counted according to the standard definition is also meaningful from a reproductive autonomy standpoint. First, according to the standard indicator, pregnant and post-partum amenorrhoeic women who are unmarried and not in a stable union are only counted by the indicator if they have had sexual intercourse in the previous 30 days, despite research showing that substantial declines in sexual activity during pregnancy and the post-partum period are common [46–48]. Second, the standard indicator assumes a woman’s demand by asking a woman about her pregnancy intention retrospectively in relation to the pregnancy she is currently carrying or her recently born baby. Past research has found this question to be unreliable in part due to fluidity in the concept of “wantedness” once a pregnancy, or birth, has taken place [49, 50]. Instead, our direct question is prospective in nature and asks pregnant women about their demand for family planning after their current pregnancy. Reframing the concept of demand in this way by asking directly about future, theoretical pregnancy, rather than a current, existing pregnancy, increases the number of women in the denominator considerably. As such, we believe that it is a more simple and direct way to measure a woman’s future fertility intentions, and thus, ultimately, her demand for family planning.

This study also sheds light on using decisional autonomy to operationalize the construct of choice in family planning service delivery. In Argentina and India, the most significant contribution to changes in the value of indicator’s numerator is the addition of the construct of choice, the incorporation of which causes the value of the indicator to decrease by slightly more than 20 and 30 percentage points, respectively [22]. In our study population, a much larger percentage of women in Argentina and India were using long-acting and permanent methods. While we cannot assess causality, this may reflect real or perceived limitations of method availability or a culture of directed counseling that reflects an implicit bias towards certain methods—either of which may ultimately limit women’s autonomous choice. In these contexts, programs may consider examining ways to strengthen service provision in a way that empowers women to achieve greater decisional autonomy to better meet the person-centered principles of “demand satisfied”.

Last, in relation to the construct of satisfaction, the standard indicator assumes that that all women without “unmet need” have a met need [27], but fails to take into account user dissatisfaction. Reasons for dissatisfaction vary, from individual-level reasons such as those associated with unwanted side effects, to those that occur at the system-level such as dissatisfaction related to user fees or other barriers [51]. Women who are unsatisfied with their contraceptive method are more likely to discontinue its use, with estimates suggesting that contraceptive discontinuation could account for one-third of unintended births [52]. A study using Demographic and Health Survey (DHS) data from 34 countries conducted between 2005–2010 found that women who had previously discontinued use of a contraceptive method and were categorized as having current unmet need for family planning accounted for 38% of total unmet need and 10% of all women surveyed [25]. In our study, accounting for women’s satisfaction with their current method reduced the value of the alternative formulation of “demand satisfied” in Argentina by ~30% and in Ghana by ~16%. Categorizing women who are dissatisfied with their method as having unmet need, even if they are using a method of contraception, has been proposed elsewhere [27]. Failure to explore and address women’s satisfaction with their current contraception method may be a missed opportunity to avert significant future unmet need—itself a construct related to satisfaction. Ironically, method dissatisfaction may drive future non-use, which is captured in the current measure as demand. This problem again illustrates ambiguity in the underlying concepts or phenomena that the indicator seeks to measure and their operationalization through the standard definition of the indicator that has clear programmatic implications. Directly including a measure of women’s satisfaction in “demand satisfied” could provide national family planning programs with a better picture of whether the contraceptives needs of women are truly being met, and do so in a way that could have real programmatic dividends in addressing unwanted fertility.

Our assessment of construct validity using regression highlights the imprecision in the standard indicator with the underlying construct of “demand satisfied” from a person-centered standpoint. The strong positive association observed between a woman’s use of her preferred contraceptive method and whether her demand was satisfied with the person-centered, alternative indicator versus those whose demand was satisfied only with the standard indicator suggests that the alternative indicator is grounded in human rights principles inherent to person-centeredness. While many factors may prevent a woman from using her preferred method, the World Health Organization recognizes that a woman’s ability to use the method she prefers is fundamental to the health of women and families and is central to ensuring that contraceptive programs are rooted in human rights principles [45].

Our study has several strengths. In testing the effects of different approaches to operationalize the fundamental concepts that this indicator aims to capture, we incorporated a range of previously validated measures to capture dimensions of the constructs satisfaction, demand, and choice and to explore variance in the results compared to the standard definition. Our iterative presentation of the indicator value by varying the underlying constructs allows examination of the contribution of each component to more person-centered estimates of “demand satisfied.” As our study sample includes a large and randomly selected population, we expect our results to be robust to a wide range of women and generalizable.

At the same time, our study has some potential limitations. To center women’s voices, we directly asked women about their sexual activity and perceived risk. We acknowledge that such responses may be subject to recall or social desirability bias, but no more than that with the standard indicator. In fact, asking about demand for contraception instead of sexual activity may lead to more accurate results in conservative settings or amongst population groups in which discussions about sexuality are sensitive; however, this should be assessed through further research. Focusing on women who state they want to use a contraceptive method in the denominator is a direct way of asking women about both risk and demand, but it does miss women who want to avoid pregnancy but do not want to use a contraceptive method for various reasons. We argue that even though our proposed measure of “demand satisfied” may miss some women at clinical risk of unintended pregnancy, these women would be unlikely to use a method even if the supply- and service-side barriers implied by “demand satisfied” indicator were addressed [27]. We also recognize the complexities associated with some women’s preferences for traditional methods versus modern methods and how that may influence our results. For example, some women may prefer non-medical or traditional methods. Should a provider steer them in the direction of a modern (and generally—but not always) more effective method, they may have lower values of decisional autonomy and satisfaction, despite using a more effective method. Here, we argue that an important tenet of person-centered care and informed decision making is to support a client in meeting their needs–even if after reviewing the pros and cons, a client wants to use a method that is less effective than other alternatives. As all contraceptive choices vary in their efficacy, real use effectiveness, and other pros and cons, decisional autonomy trusts women to make the decisions that are right for themselves, given adequate information and support to choose. While our study used random sampling to increase generalizability, the demographic characteristics of our population show that only a small percentage of our sample in India was categorized in the poorest wealth quintile nationally, thus our sample may not be representative of the general population of India due to district-level variation. Similarly, we find some other slight differences in our study population with regard to age structure and literacy. However, as the goal of this analysis was not to provide nationally generalizable estimates of the indicators, but rather to comparatively asses how the indicators perform within a given population, demographic differences between our study population and the general population do not impact our findings. Finally, we only surveyed women aged 15–49 years, in keeping with the current definition of women of reproductive age, and thus we potentially missed some women who may have unmet need for family planning who fall outside of that age range.

In conclusion, our study suggests that the percentage of women with “demand satisfied” for family planning after incorporating the person-centered constructs of demand, choice, and satisfaction is substantially lower than that obtained using the standard indicator in Argentina and India but remains approximately the same in Ghana. The dramatic change in the value of the indicator is attributable to strengthening the representation of constructs related to personal choice and satisfaction but also expanding the construct of demand to include all women who indicated that they want modern contraception, and including women based on their stated demand for family planning, rather their marital status or sexual activity. In Ghana, unlike Argentina and India, there was little change in the indicator value when incorporating dimensions of choice and satisfaction, suggesting that the care received by most women in Ghana may already reflect these dimensions of person-centered care. That said, even in Ghana our regression results highlight profound differences in outcomes between women who have their “demand satisfied” per the standard indicator but not per the alternative definition incorporating individual perspectives. Further research is warranted to examine indicator performance between the standard and alternative indicator formulations may provide insight into whether there are systematic differences in women who receive person-centered care to meet their demand for contraception versus those who do not, with potentially valuable lessons about equity.

The results of this study have implications for person-centered, respectful delivery of contraceptive counseling and services to improve the dimensions of choice and satisfaction and more accurately reflect demand for contraception. While we recognize that adding additional questions to global surveys as would be required by our proposed measure may be challenging from a feasibility perspective, we believe that our study provides an important starting point for research focused on both simplifying and reframing. The large number of survey questions required and the burdensome analysis needed to compute the standard “demand satisfied” indicator are recognized drawbacks. Moreover, incorporating individual perspectives into a widely collected measure for tracking population progress toward FP2030 goals may help elevate person-centered quality of care as well as effective coverage.

## Supporting information

S1 File Inclusivity in global research checklist. (DOCX)

S1 Checklist Human subjects research checklist. (DOTX)
